# Interpersonal influences on decision-making capacity: a content analysis of court judgments

**DOI:** 10.1093/medlaw/fwad017

**Published:** 2023-06-09

**Authors:** Kevin Ariyo, Nuala B Kane, Gareth S Owen, Alex Ruck Keene

**Affiliations:** Mental Health, Ethics and Law Research Group, Department of Psychological Medicine, Institute of Psychiatry, Psychology and Neuroscience, King’s College London, UK; Mental Health, Ethics and Law Research Group, Department of Psychological Medicine, Institute of Psychiatry, Psychology and Neuroscience, King’s College London, UK; Mental Health, Ethics and Law Research Group, Department of Psychological Medicine, Institute of Psychiatry, Psychology and Neuroscience, King’s College London, UK; Mental Health, Ethics and Law Research Group, Department of Psychological Medicine, Institute of Psychiatry, Psychology and Neuroscience, King’s College London, UK; Dickson Poon School of Law, King’s College London, UK

**Keywords:** Capacity, Court of Protection, CRPD, Inherent jurisdiction, Psychiatry, Undue influence

## Abstract

For many purposes in England and Wales, the Court of Protection determines whether a person has or lacks capacity to make a decision, by applying the test within the Mental Capacity Act 2005. This test is regularly described as a cognitive test with cognitive processes discussed as internal characteristics. However, it is unclear how the courts have framed interpersonal influence as negatively impacting upon a person’s decision-making processes in a capacity assessment context. We reviewed published court judgments in England and Wales in which interpersonal problems were discussed as relevant to capacity. Through content analysis, we developed a typology that highlights five ways the courts considered influence to be problematic to capacity across these cases. Interpersonal influence problems were constructed as (i) P’s inability to preserve their free will or independence, (ii) restricting P’s perspective, (iii) valuing or dependence on a relationship, (iv) acting on a general suggestibility to influence, or (v) P denying facts about the relationship. These supposed mechanisms of interpersonal influence problems are poorly understood and clearly merit further consideration. Our typology and case discussion are a start towards more detailed practice guidelines, and raise questions as to whether mental capacity and influence should remain legally distinct.

## I. INTRODUCTION

Whether and how the law can take account of interpersonal influences on decision making is an issue that has exercised law-makers, practitioners, and scholars for several years. What is sometimes lacking in these debates, however, is empirical evidence. Covering the first 12 years after the Mental Capacity Act (MCA 2005) was implemented, we outline the results of a qualitative content analysis of Court of Protection cases and other legal cases in England and Wales, shedding light on three key questions:

- What are the common characteristics of mental capacity court cases where interpersonal influence problems are a concern?
- What are the different categories of interpersonal influence problems on decision-making capacity?
- How do the courts discuss interpersonal influence problems using the language of the functional test in the MCA 2005?

## II. BACKGROUND

Many jurisdictions have established a legal framework to decide whether a person is capable of making specific decisions for themselves, based on the functional model of ‘decision-making capacity’. Simply put, these frameworks set out provisions focusing on two core ethical duties: to safeguard personal autonomy in making decisions and to protect vulnerable people from experiencing harm.^1^ Under the functional model of capacity, the person assessing capacity must make careful inferences as to whether a person has the psychological abilities needed to make the decision, without discriminating against the person’s values and motivations, or imposing their own.^2^ Capacity assessments can take place in any context, with a health or social care professional being the most likely to undertake a formal assessment.

Applying such a model to more complex decisions can be difficult. In England and Wales, the MCA 2005 requires that the assessor (or the court) be satisfied that the person is unable to understand, retain, use, or weigh information relevant to a specific decision, or to communicate their decision.^3^ This functional inability must be caused by the impairment of mind or brain, and the importance of identifying the ‘causative nexus’ between impairment and functional inability was made clear by the Court of Appeal in *PC v NC v City of York*. ^4^ The Court noted that starting with impairment, as opposed to potential inability, gave rise to the danger that ‘the strength of the causative nexus between mental impairment and inability to decide is watered down’.^5^ Emphasizing the need for a causative link, the Court identified that phrases, such as any inability being ‘referable to’ or ‘significantly relates to’ the impairment in question, were inadequate.^6^

One criticism of the functional model enshrined in the MCA 2005 is that these legal criteria encourage assessors to consider the test as a ‘cognitive’ one,^7^ and to locate decision-making abilities (and in particular, inabilities) as purely internal characteristics. Models of relational autonomy reject this approach, by asserting that all aspects of self, and therefore autonomy, are shaped by interactions with others. For example, Camillia Kong has argued that the MCA 2005 has an overly individualistic focus on characteristics such as the person’s own values, wishes, preferences, and diagnosis or diagnoses.^8^ While internal characteristics are important, several social psychology studies have demonstrated—under empirical conditions—that social stimuli are fundamental to the decision-making process.^9^ Our emotions, cognitions, and behaviours are susceptible to information disclosed by other people (informational social influence), as well as to decisions that we witness other people making (normative social influence), particularly when we are feeling uncertain.^10^ The *relative* impact of these processes varies from person to person and are poorly understood in ‘vulnerable adults’, a term used by the courts among others.^11^ Of course, it should not always be assumed that social influence is benign. So, if the MCA 2005 is, indeed, too individualistic, it may not adequately recognize the importance of freedom from duress and coercion in a person reaching an autonomous decision.^12^ This would raise further questions as to how judges handle relational concepts within a capacity framework in the absence of any formal criteria or guidance.

The relational aspect identified above is perhaps most evident where the person is in a situation where one or more other people appear to be exploiting or in some other way exposing them to harm. In such a situation, decision-making capacity as embodied in the MCA 2005 seems to be only part of the problem. The Law Commission, in its project leading to the MCA 2005, had grappled with the fact that such a situation could require a bespoke solution, having noted that:

there are other people who are not incapable of taking their own decisions, but are also especially vulnerable to abuse or neglect from which they are unable to protect themselves. Some machinery is needed to protect them and the existing procedures outlined in this review already do so to some extent. However, they are widely believed to be unsatisfactory for this purpose.^13^

These concerns were not taken forward by the government when legislating, but judges have, however, crafted their own solution to the problem. In the absence of any statutory provision, these steps have been taken under the High Court’s inherent jurisdiction, described elegantly by Lord Donaldson as the ‘great safety net’ filling in the gaps of the statutory law.^14^ Prior to the passage of the MCA 2005, this safety net was required to secure the interests both of those who would now be seen as lacking decision-making capacity, and those who *have* such capacity but whose decision-making capacity is compromised by the actions of a third party.^15^ Subsequent to the passage of the MCA 2005, there was some doubt as to whether the Act had entirely delineated the scope of those to whom the courts could afford protection.^16^ However, in 2012, the Court of Appeal confirmed that the passage of the MCA 2005 did not entirely fill the gaps within the ‘great safety net’ described by Lord Donaldson, and therefore it would have been inappropriate to remove the ability of the High Court to take protective steps by using the inherent jurisdiction in relation to an adult even where they have decision-making capacity.^17^

Importantly, however, a High Court judge can only exercise this jurisdiction if the person in question *has* capacity under the MCA 2005 to take the decision in question; for instance, whether to remain in contact with someone who appears to be abusing them. This means that they must first consider whether the person should be considered to lack capacity, thereby, directly applying the MCA 2005. The development of the inherent jurisdiction has been subject to academic criticism, not least because it is constrained by no governing statutory principles,^18^ but its continuing growth seems to reflect a need experienced by health and social care professionals who are confronted by patients in situations at the margin of the MCA 2005.^19^

In a context where statutory reform did not take place to address the limitations identified by the Law Commission in the 1990s,^20^ we sought to examine how judges have been approaching interpersonal factors when considering the concept of capacity as now defined within the MCA 2005. In approaching this question empirically, we aimed to provide a *psychological* account of these relational processes as they are referred to in court judgments. Our account focuses on ‘interpersonal influence’, defining ‘interpersonal influence problems’ as any action or characteristic of another person that was constructed in legal submissions to have potentially had a deleterious effect on P’s decision-making capacity. We excluded cases where interpersonal influence was only constructed as positive, as this would have required a more complex search and the literature on supporting capacity is relatively better developed.^21^ We chose to focus on ‘interpersonal influence’ rather than ‘undue influence’ as this better enabled us to explore in detail how the courts considered the relational context to be operative on decision-making capacity.

Relational factors considered by the courts are not always described as ‘undue influence’, especially in cases where it is likely from the outset that P (the subject of proceedings before the court) lacks capacity and, therefore, would not require intervention under the inherent jurisdiction. By avoiding this technical term, we sought to make our search sensitive to a wider range of relevant evidence, as well as a wider range of discussion from judges on decision-making capacity. Furthermore, ‘undue influence’ presupposes that the relational context is one of harmful intentions, when the relational context may be better described as one of high interpersonal pressure, regardless of intentions. We were also aware that the term ‘undue influence’ has developed a life of its own in contexts less relevant to our study; for instance, in relation to looking back after the event to determine whether a gift was made voluntarily.^22^

To investigate interpersonal influence problems, we undertook a review and qualitative content analysis of Court of Protection cases and other legal cases in England and Wales in which both interpersonal influence problems and decision-making capacity were considered, and in which a determination was reached as to the individual’s decision-making capacity.

## III. METHODS

### A. Search criteria

The search strategy was adapted from the criteria used in a previous, large-scale review of capacity judgments in the Court of Protection (CoP).^23^ This study identified all published judgments from England’s CoP or Court of Appeal [Civil Division] (CoA) cases on appeal from the CoP, available on Westlaw and BAILII databases as of 11 September 2018; a total of 407 CoP and 26 CoA judgments. From these, we selected adult cases (where P was over 18 years old)^24^ that contained rationales for judgments of incapacity or intact capacity of P in relation to a specific decision; a total of 131 judgments—128 CoP and three CoA judgments. For the purposes of the present study, we carried out an update search of Westlaw on 4 November 2019, resulting in a further 16 CoP and one CoA judgments containing capacity rationales. This gave a total of 144 CoP and four CoA capacity judgments.

As we were interested in all judgments considering both capacity and interpersonal influence problems, we then identified (via Westlaw) all published High Court (non-COP) judgments, as of 4 November 2019, which invoked the inherent jurisdiction, using the search terms ‘coercion’, ‘undue influence’, and ‘inherent jurisdiction’. This search returned 15 published judgments. From these, we selected judgments for which the capacity of P was considered; a total of Four High Court (non-COP) judgments.

The 152 selected judgments were screened for references to interpersonal influence as impairing or not impairing P’s decision making in relation to the capacity issue before the court, and cases without such references were excluded. We considered only descriptions of actual influence by a named influencer, not P’s hypothetical vulnerability to influence, because we wanted to examine *how* the courts approached P’s ability to make actual decisions (for instance, contact with the influencer). Nor did we address situations in which the court did not, in fact, reach a conclusion as to P’s capacity. A final sample of 20 judgments (15 CoP, two CoA, and three non-COP High Court cases) met these criteria.

### B. Data analysis

All authors are researchers in the Mental Health and Justice (MHJ) project, which takes an interdisciplinary approach to understanding mental capacity. K.A. has a background in psychology and drew insights from social and cognitive psychology to understand interpersonal dynamics. N.K. is a practising psychiatrist with experience in analysing CoP judgments on capacity. A.R.K. is a practising barrister and legal academic. G.O. is an academic psychiatrist with a research focus on mental capacity.

K.A. and N.K. managed the full dataset of cases using Nvivo 12. K.A. first coded cases for descriptive characteristics such as age and gender of P, the capacity issue before the court, P’s impairment of the mind or brain, and the judge’s determination on P’s capacity.

Qualitative content analysis (QCA) was then used to analyse the sample through an iterative coding process.^25^ QCA is inherently qualitative as codes are generated from the interpretation of the data and are then applied to the data via close reading. It also has a quantitative component as codes are counted in order to detect patterns in the data which might guide further interpretation. It has been argued that content analysis of court judgments is particularly useful to gain knowledge of how judicial decisions are justified.^26^

K.A. read through the sample of 20 judgments, independently coding references to interpersonal influence, from a named influencer, as impairing or not impairing P’s decision making in relation to the matter before the court. The independently coded references were then discussed by K.A. and N.K. to reach a consensus on whether they met the definition of interpersonal influence. Disagreements were resolved by appealing to G.O., who arbitrated and made the final decision. This resulted in a total of 54 references.

These references were then read by K.A. and N.K. and a provisional coding scheme for *types of influence on decision making* was developed. K.A. and N.K. then proceeded with an iterative coding process, independently applying these codes to the whole sample, and refining the codes and resolving key boundary issues through discussion, with final arbitration from G.O. to resolve remaining disagreements. This resulted in a final coding scheme consisting of five types of influence problems on decision making and a residual category called ‘no specific mechanism’. We then carried out a reliability check using a ‘fuzzy kappa’ statistic, which measures the degree of agreement between the two coders in applying the pre-established coding scheme to the dataset, and adjusts for the fact the categories are non-exclusive (ie, that more than one category could be applied to a piece of text). This resulted in an intercoder agreement statistic of 0.80 across the five types,^27^ suggesting good interrater reliability for the typology categories.

K.A. subsequently examined references for links to sections 2 and 3 of the MCA 2005,^28^ including linkage to abilities (understanding, retaining, using or weighing, communicating) and to the causative nexus (inability to make a decision *because of* an impairment of the mind or brain). Finally, we used the themes generated by our qualitative content analysis as a prompt for further case-based analysis.

## IV. RESULTS

### A. Sample characteristics

As outlined in Table 1, our final sample included 20 court judgments, containing 54 statements pertaining to interpersonal influence problems on capacity from a named influencer. Most of these judgments were derived from either the CoP (16 cases) or the CoA following an appeal from the CoP (two cases). The remaining judgments were derived from the High Court exercising its inherent jurisdiction (three cases). Interestingly, only two cases in the sample were decided prior to *PC v NC v City of York Council.*^29^

**Table 1.** Characteristics of each judgment in the final sample

Name of the case	Neutral citation	MM/YY	Type of decision(s)	Material impairment or disturbance of mind or brain	Outcome(s)	N^a^
*PCT v P, AH, & A Local Authority* [2009]	COPLR Con Vol 956	12/09	Care, contact, general activities, treatment	Intellectual disability	Lacked capacity to take all decisions	2
*A Local Authority v Mrs A and Mr A* [2010]	EWHC 1549 (Fam)	06/10	Contraception	Intellectual disability	Lacked capacity to take the decision	5
*D v R (Deputy of S) and S* [2010]	EWHC 2405 (COP)	07/10	Contact, contract, property/finances	Dementia	Lacked capacity: property/finances	2
					Unclear otherwise	
*PC v NC v City of York Council* [2013]	EWCA Civ 478	05/13	Contact	Intellectual disability	Appeal upheld	2
			Resumption of married life			
*LBX v K, L and M* [2013]	EWHC 3230 (Fam)	06/13	Care, contact, residence	Intellectual disability	Had capacity to take all decisions	1
*A Local Authority v WMA* [2013]	EWHC 2580 (COP)	07/13	Care, contact, residence	Autistic Spectrum Disorder	Lacked capacity to take all decisions	1
*London Borough of Redbridge v G & Ors* [2014]	EWCOP 485	02/14	Contact, residence, property/finances	Dementia	Lacked capacity to take all decisions	4
*NCC v PB and TB* [2014]	EWCOP 14	03/14	Care, contact, residence	Anxiety	Lacked capacity to take all decisions	7
Re MRJ (Reconsideration of Order) [2014]	EWCOP B15	04/14	Revoke a lasting power of attorney	Dementia	Lacked capacity to take the decision	1
*Derbyshire County Council v AC* [2014]	EWCOP 38	10/14	Care, contact, residence	Intellectual disability	Had capacity: sexual relations	1
			Sexual relations		Lacked capacity: care, contact, residence	
*Re A* [2016]	EWCOP 3	01/16	Contracts, property/finances	Schizophrenia	Lacked capacity to take all decisions	1
*Newcastle Upon Tyne City Council v TP* [2016]	EWCOP B4	11/16	Care, property/	Intellectual disability	Lacked capacity to take all decisions	1
			Finances, residence, treatment			
*London Borough of Brent v NB* [2017]	EWCOP 34	10/17	Care, contact, general activities, treatment	Intellectual disability, brain injury	Had capacity: general activities, contact	5
					Lacked capacity: care	
*Re B* (Capacity: Social media: Care and Contact) [2019]	EWCOP 3	02/19	Care, contact, property/finances, residence, sexual relations, social media	Intellectual disability	Had capacity: residence	5
					Lacked capacity: care, contact, property/finances, sexual relations, social media	
*Southend-On-Sea Borough Council v Meyers* [2019]	EWHC 399 (Fam)	02/19	Residence	None reported	Had capacity to take the decision	4
*London Borough of Hackney v SJF & Anor* [2019]	EWCOP 8	03/19	Care, contact, contracts, residence, treatment	Intellectual disability	Lacked capacity to take all decisions	4
*B v A Local Authority* [2019]	EWCA Civ 913	06/19	Care, contact, residence, sexual relations, social media	Intellectual disability	Appeal dismissed	1
*University Hospitals Bristol NHS Trust v RR* [2019]	EWCOP 46	08/19	Treatment	Emotionally unstable personality disorder, Asperger’s syndrome	Lacked capacity to take the decision	1
*London Borough of Croydon v KR & Anor* [2019]	EWHC 2498 (Fam)	09/19	Residence	Brain injury	Had capacity to take the decision	3
*Redcar & Cleveland Borough Council v PR* (No 2) [2019]	EWHC 2800 (Fam)	09/19	Contact, residence	None reported (previous ‘mental health difficulties’ acknowledged)	Capacity to take all decisions	3

a N is the number of statements that were coded to any category of interpersonal influence.

**Table 2.** Outlining how the courts link interpersonal influence to the language of the functional abilities in the MCA (2005)

Functional ability	Inability to preserve independence and free will	Restricting P’s perspective	Valuing or dependence	Suggestibility	Denial of facts	Causative Nexus	No mechanism	Total (inc. duplicates)
Understanding	0	1	1	1	0	0	1	4
Retaining	0	1	0	0	0	1	0	2
Use or Weigh	3	6	4	2	1	5	1	22
Total (inc. duplicates)	3	8	5	3	1	6	2	

A range of conditions were considered to be the material ‘impairment of mind or brain’ in these cases. P was diagnosed with an intellectual disability in almost a half of cases in our sample (10 cases), with the severity of learning difficulties varying from ‘mild’^30^ to ‘moderate’,^31^ but none recorded as meeting the criteria for ‘severe’. Otherwise, the sample included various psychiatric conditions (anxiety, schizophrenia, and personality disorder), neurodevelopmental conditions (autistic spectrum disorder and Asperger’s syndrome), and neurological conditions (dementia and brain injury). In two High Court cases, there was no clear report of any current impairment of mind or brain. P was described as a vulnerable adult in both cases,^32^ and they were reported to have had capacity to take each decision.

The most common decisions in question were capacity to take decisions regarding residential matters (12 cases), contact (13 cases), care arrangements (10 cases), medical treatment, and property or financial affairs (five cases each). Less frequently, P was assessed for capacity to take decisions around sexual relations or contractual matters/testamentary capacity (three cases each), social media use and general activities (two cases each), and contraception, resuming married life, and revoking a Lasting Power of Attorney (one case each).

Of the 54 statements pertaining to interpersonal influence on capacity from a named influencer, most of the statements were used as a rationale for P not having capacity (24 statements, 44.44%) or did not explicitly state whether it was arguing for or against the person having capacity (20 statements, 37.04%). Other references were used as a rationale for P having capacity (five statements, 9.26%), or P having capacity except in specific situations (six statements, 11.11%).

### B. Types of interpersonal influence problems

We found five main types of interpersonal influence problems from our sample.^33^

#### 1. Inability to preserve independence and free will

Fifteen references; eight cases:

I was advised that AC has ‘difficulty in sustaining an independent view’ if she is exposed to an alternative view from someone who ‘is influential’ such as her mother, or boyfriend.^34^

This was the most referenced theme. Some of these statements questioned whether P was able to preserve their individual autonomy when exposed to the influencer. The theme also included references to free will or an overwhelming of P’s ability to make true, autonomous, voluntary choices. There may have been a description of an overwhelming power dynamic or a comment that P was unable to resist or free themselves from the influence. Perhaps unsurprisingly, at least one reference was coded as such in each of the three non-COP inherent jurisdiction cases.

One relevant case included *Derbyshire County Council v AC*.^35^ The subject of proceedings, AC, was a 22-year-old woman who had a ‘significant learning disability’,^36^ with some preserved independent living skills but limited executive functioning^37^ and comprehension. Partly considering her history of abusive relationships, the local authority raised concerns over her living occasionally with her boyfriend who had prior convictions for domestic violence. AC had appeared to fluctuate between wishing to live with her parents and her boyfriend, sometimes ‘within the course of the same conversation’.^38^ In relation to AC’s capacity to take a decision on residential matters, the solicitor acting on behalf of the local authority submitted that ‘AC has “difficulty in sustaining an independent view” if she is exposed to someone who “is influential” such as her mother, or boyfriend’.^39^ Cobb J decided, in the interim, that AC lacked capacity to take a decision regarding her residence until such a time in the future where her capacity would be re-assessed. It is unclear to what extent AC’s inability to preserve her independence and free will contributed to the finding that she lacked capacity, though Cobb J’s emphasis on future capacity assessments suggests that removing the alleged influencers could be key to AC’s prospects of regaining capacity.

The issue of independence was also contested in *Southend-On-Sea Borough Council v Meyers*.^40^ Mr Meyers (who waived anonymity) was at the time of proceedings a 97-year-old care home resident. He had capacity to take a decision on his residence, though he was considered a vulnerable adult due to his blindness and the conduct of his son and carer, KF. The issues raised in relation to KF concerned his alleged neglect and abuse of Mr Meyers and a history of intimidating behaviour towards social care services. The local authority had made several prior attempts to support Mr Meyers with his care needs over a 2-year period, with very limited success. It became clear that Mr Meyers’ physical health was deteriorating amid reports that his house was severely lacking in basic amenities. Mr Meyers himself was malnourished and required hospital treatment, to the point where his life was at risk. After feeling that they had met their legal obligations, the Local Authority sought an injunction.

The relationship between Mr Meyers and KF was a key point during the discussion, which focused on Mr Meyers’ decision-making abilities and whether to invoke the inherent jurisdiction to safeguard him. At the time, Mr Meyers had wished to live with his son, despite having stated the opposite only months previously. The expert social worker submitted that Mr Meyers could ‘articulat[e] his wishes and feelings autonomously and not under influence or coercion’.^41^ However, this did not convince Hayden J. His judgment referred to the ‘intensity of this relationship [which] occludes Mr Meyers’ ability to take rational and informed decisions’,^42^ and that the consequence of this influence ‘is to disable Mr Meyers from making a truly informed decision’.^43^ On this basis, and due to the physical risks to Mr Meyers, Hayden J exercised the inherent jurisdiction to allow Mr Meyers to return home as he wished, but prevent his son from residing with him.

#### 2. Influence restricting P’s perspective

Twelve references; seven cases:

the enmeshed relationship that he has with AH which severely restricts his perspective in terms of being able to think about his future.^44^

In this situation, the influencer or the relationship with the influencer impaired P’s decision-making process due to confusion/anxiety or restricted P’s ability to consider abstract options or future possibilities. P’s perspective was therefore narrowed because of the influence.

Considering future possibilities was a prominent theme in *PCT v P & Ors*.^45^ P was a young man who had been diagnosed with severe epilepsy and a ‘lower end of mild’ learning disability,^46^ both of which were considered to impact his functioning. His mother, AH, had cared for P for 18 years until recently, when concerns were raised about their decision for P to discontinue his epilepsy medication, as well as the subsequent deterioration in P’s condition and daily living skills. On assessment, P was found to lack capacity to take a range of decisions relating to his health, contact and welfare. Hedley J appeared to refer heavily to the alleged impact of AH on P’s ability to use abstraction in the decision-making process, using terms such as ‘think about his future’ and ‘visualise any prospect’ as justification of P’s functional inability.^47^ Hedley J was, however, keen to stress that these factors only led P to lack capacity when considered in addition to his impairments and their effects on his functional ability.

In other cases, the influence was described as having introduced confusion or otherwise complicating the decision-making process. There were some notable examples of confusion triggering court proceedings, even when P was considered to be above the threshold of capacity at the beginning of proceedings. For example, in *London Borough of Redbridge v G & Ors*,^48^ the local authority initially advanced proceedings to protect a 94-year-old woman (G) under the inherent jurisdiction, as they considered her to have capacity to take decisions regarding her contact, residence and property, and affairs. The reason for the injunction was due to concerns that her carers, C and F, were financially exploiting her and compromising her ability to make informed decisions. On G’s part, she did not wish to lose the independence of living in her own home and feared that without C and F or anyone else to care for her, she would be placed in a care home against her wishes. The local authority had made previous attempts to communicate that they would provide sufficient support for G to remain in her own home, but suspected that C and F were withholding this information from G and exaggerating the risk of G’s removal so as to increase her dependency on them.^49^

Despite the initial finding of G having capacity, the court sought evidence from independent experts, which led to a ruling that G did not have capacity. The expert evidence is of interest partly because of the different reasons provided for G not having capacity. The social worker argued that this was because of the undue influence, whereas the psychiatrist acknowledged the influence as undermining capacity but considered P to lack capacity because of a mild-to-moderate dementia. Ultimately, the judge preferred the evidence of the psychiatrist, partly on the basis that the psychiatrist was qualified to recognize mental impairments. Nonetheless, the judge described the influence of C and F as having ‘compromised’ decision making, rendering it ‘even more difficult for G’ and that she was ‘at times almost paralysed by the threats regarding her removal to a care-home or to have F take over her personal and intimate care’.^50^ It is important to consider that, while it was not contested that P was both vulnerable and subject to extreme social pressure, the course of proceedings depended entirely on whether P had a formal impairment of mind or brain.^51^

#### 3. Valuing or being dependent on the relationship

Twelve references; nine cases:

Perhaps I do place more importance or weight on ST’s wishes and feelings but I think this is only normal, as she is my wife. I want to have the choice to live my life as I want.^52^

‘She fails the third test of capacity because her decision making processes are heavily influenced and distorted by her overwhelming sense of loyalty to JT and KT and her need to retain these important relationships for her’.^53^

In these statements, the influence was considered to show itself through the value P placed on the relationship with the influencer, which resulted in P placing particular weight on the perceived views of the influencer or acting to preserve the relationship at all costs. Unlike the following theme, suggestibility, the evidence here focused on factors that were specific to the relationship between P and the influencer. This took place in either a marriage, familial or caring context. In all these cases, there were clear tensions between the duty to protect and the freedom to make (capacitous) unwise decisions, as well as the right to respect for one’s private and family life under Article 8 of the European Convention on Human Rights.

Some statements emphasized that it is common for close relations to have a strong input into one’s decisions. For example, in *London Borough of Croydon v KR & Anor*,^54^ a man was assessed to have capacity to take a decision as to where he lived and whom he lived with. Because there was no dispute that KR had capacity, he did not fall within the scope of any protections under the MCA 2005. In the light of concerns that he was being unduly influenced by his wife, the only safeguarding option would have been to invoke the inherent jurisdiction of the High Court. KR responded to those concerns by stating that he considered this to be ‘normal, as she is my wife’.^55^ The judge decided not to intervene on this basis, having argued that:

it is important to be careful to distinguish between the entirely natural and common influence that one close family member will have over another, and the “undue influence” or “coercion” identified in SA and DL.^56^

This echoed a similar argument in *London Borough of Hackney v SJF & Anor*,^57^ which involved a woman with mild learning disability. When asked to consider how SJF’s relationship with her son may impact her capacity to take a decision as to where to live, SJF’s solicitor opined that her ‘clearly stated wishes simply give greater weigh[t] to her family life and her wish to be with her son, in her own home of many years’,^58^ despite her son’s challenging behaviour towards her. However, this argument did not persuade Senior Judge Hilder to find that SJF had capacity. Instead, she preferred the evidence of the consultant psychiatrist, who argued that it was SJF’s learning disability that led her to be unable to understand, retain, or use or weigh information about her treatment needs and risks to returning home with her son.

On the other hand, some statements seemed to suggest an unequal relationship between P and the alleged influencer. P’s dependence on this person could then be reasoned to affect their capacity. In *Re MRJ*,^59^ the CoP heard an application with respect to an 82-year-old woman with dementia, amid concerns of emotional and financial abuse from her daughter and grandson. When providing evidence as to whether MRJ could revoke her existing Lasting Power of Attorney, the consultant psychiatrist successfully argued that MRJ was unable to use or weigh information relevant to the decision as her decision-making processes were ‘heavily influenced and distorted by her overwhelming sense of loyalty’ and ‘her need to retain these important relationships for her’.^60^ Another example is *A Local Authority v WMA*,^61^ in which a 25-year-old man with autism spectrum disorder was alleged to have experienced long-term neglect. WMA was cared for by his mother, MA, and both opposed an increase in intervention from care services. The court ruled that WMA was unable to use or weigh information relevant to deciding where he should live, and Cardinal J’s observations on capacity focused heavily on WMA’s relationship with his mother, with whom WMA had an ‘unhealthy degree of interdependence’.^62^ It is notable that in both cases the judges regarded the close family member(s) as having contributed to P’s social isolation and obstructed potential social support. Cardinal J went so far as to rule that it was in WMA’s best interests to move away from living with his mother, in part because this may reduce his dependence and allow him to form relationships with others.

#### 4. Suggestibility

Six references; five cases:

her inability to understand the actions of others (for example, fellow patients who stole from her or induced her to pay more than the real value of items) and her extreme suggestibility (I believe that I could have induced her to make over all her assets to me in return for helping her to meet with a celebrity), I consider that A is extremely vulnerable to exploitation and abuse.^63^

In these statements, the interpersonal influence was thought to be problematic through P’s tendency to align their decisions with the wishes of others. The underlying process has been described as suggestibility—a tendency to acquiesce, or an eagerness to please. Although our sampling frame selected cases that discussed influence from a named person, within these discussions of suggestibility we also found references to an abstract, or trait-like, suggestibility that was not specific to that named person. These references suggest a view that P’s decision making is too malleable because of their impairment of the mind or brain, as opposed to P only being made vulnerable from the deliberate actions of a specific person or being placed in an extreme environment. The implication was that a certain level of influence from anyone is more problematic for some people than others, in terms of their ability to make capacitous decisions.^64^

For example, *Re A* concerned a 78-year-old woman with schizophrenia who was subject to a family dispute with regards to who should be appointed deputy for her property and affairs.^65^ It is not clear whether A enjoyed positive relationships with all the family members involved. When concluding that A did not have capacity to manage her own affairs (and hence giving the court the jurisdiction to appoint a deputy to manage them on her behalf), the consultant psychiatrist appeared not to justify this with reference to factors specific to her family relationships. Instead, he pointed to past scenarios in which A was exploited by various third parties, and then to a hypothetical scenario in which he believed he could exert malign influence on A’s decision making that would lead her to lose all her assets. With reference to her diagnosis of schizophrenia, he argued that this rendered A widely vulnerable to being financially exploited by others *in general*.^66^

We identified similar patterns in the other cases, such as *London Borough of Brent v NB*.^67^ These proceedings concerned a 22-year-old man, MB, who was diagnosed with a learning disability and an acquired brain injury, and he had a physical disability due to cerebral palsy. MB generally found it difficult to communicate his wishes, and although it was accepted that he did not wish to undergo a residential placement for additional support, his capacity to take various decisions was in question. The assessment was extensive, in part because of MB’s support needs and the role of his mother, NB, in his care. While the judgment focused primarily on the maternal relationship, which was described throughout as ‘enmeshed’^68^ it was also discussed whether MB’s decision making could be compromised by non-specific third parties. For example, the expert clinical psychologist ‘noted that [MB’s] opinion changed rapidly and considered that he was vulnerable to the influence of others in making his decisions’.^69^ This submission was used as evidence that MB did not have capacity to take a decision to appoint a case manager and who that case manager should be.

#### 5. Denial of facts about the influencer or the relationship

Six references; three cases:

she has shown herself to be unable to accept the fact of Mr C’s convictions (she has been told by five different professionals on seven separate occasions about these), and has been repeatedly dismissive of attempts to ‘educate’ her as to the convictions and their implications.^70^

In this, the least cited category, the rationale was that P did not have capacity because they denied negative facts about the influencer, or declined either to examine certain parts of the influencing relationship or to incorporate information relevant to those facts into their decision making where it was relevant to do so.

Most of these statements derived from *Re B (Capacity: Social Media: Care and Contact)* and the subsequent Court of Appeal decision in the same case, *B v A Local Authority*.^71^ This complex case concerned a 31-year-old woman with learning disabilities and arose in part due to safeguarding concerns with respect to a man she had met, Mr C, as well other men she had met on social media. The local authority was particularly concerned about the risk Mr C posed to her, given his history of sexual offences, his wish to marry Miss B, and recorded correspondence between them which suggested controlling behaviour. As outlined in the quote above, Cobb J concluded that Miss B could not use or weigh information relevant to deciding whom she should have contact with, in part because she seemed not to believe Mr C’s prior convictions or factor this into her decision. It was noted that the relevant information for the purposes of a decision to contact, includes ‘the positive and negative aspects of having contact with each person’,^72^ and Theis J elaborated that ‘this will necessarily and inevitably be influenced by [P]’s evaluations. His evaluations will only be irrelevant if they are based on demonstrably false beliefs’.^73^ In Miss B’s case, it would seem that her evaluation of the risk around Mr C, based on the apparent (false) belief that he had no prior convictions, rendered her unable to use or weigh the negative aspects of contact with him. No clear link was made in the judgment between her beliefs and her learning disability.^74^

Many of the same issues arose in another, earlier, case *PC v NC v City of York Council*.^75^ Here, PC was a 48-year-old woman with mild learning disabilities, who was cohabiting with her husband of 7 years (NC). NC had prior convictions for serious sexual offences and in anticipation of his release in 2011, the local authority asked whether PC had capacity take a decision to cohabit with her husband. On giving evidence, the consultant psychiatrist judged that PC was incapable of understanding or using or weighing with respect to her decision to have contact with NC, in large part because she ‘could not, or would not, accept the guilt, or even the possible guilt, of NC in respect of the matters of which he had been convicted’.^76^

Interestingly, although the first instance judge, Hedley J, agreed that the fact of NC’s guilt in relation to those offences was potentially highly relevant to PC’s capacity to take decisions in relation to his care, contact, and residence, he rejected the characterization of the issue as being whether she accepted NC’s guilt. By Hedley J’s reasoning, PC should have been entitled to make a potentially unwise judgment as to whether NC was guilty. Thus, Hedley J argued that the relevant issue was PC’s ‘learning disability, her unwillingness to examine the issue of his [NC’s] guilt, and her overwhelming desire to re-establish that relationship, and that that derives in significant part from her impairment’.^77^ By doing so, Hedley J seemed to confirm that PC merely needed to engage with this information on some level to have capacity, and that she was unable to do so.

Hedley J then ruled that it was in PC’s best interests to cohabit with NC, provided that the local authority could monitor their progress. This decision was made on the basis that PC had made a capacitous decision to marry NC, and that it would have been an intrusion upon their marital contract if they were forced to live apart. However, on PC’s appeal, the CoA overturned Hedley J’s judgment, as the psychiatrists’ evidence was considered insufficient to prove that PC’s learning disability had caused her to lack capacity. By doing so, the CoA appeared to suggest that the causative nexus argument should have been made based on PC’s learning disability alone, rather than in conjunction with PC’s examination of NC’s guilt, which was not considered to be relevant. The CoA instead acknowledged that NC was convicted and imprisoned when PC had taken a capacitous decision to marry him, and therefore their privacy should be respected as there was nothing to suggest that her impairment had worsened since then.

### C. Interpersonal influence: relation to functional abilities

In total, 19 statements from 12 cases explicitly linked interpersonal influences to functional abilities (see Table 2). These were most often made in reference to the ‘use or weigh’ criterion, followed by the understanding criterion. Only one statement made a clear reference to the presence of an influencer affecting P’s ability to retain information. The types of interpersonal influence constructed in these statements were spread relatively uniformly across the functional abilities, with two exceptions. We found evidence of ‘use or weigh’ but not understanding in the ‘inability to preserve independence and free will’ and in the ‘causative nexus’ categories. There were no clear mentions of interpersonal influence with respect to the ‘communication’ criterion: the ability to express a choice.

### D. How do judges use evidence of influence and impairments?

Fifteen references; nine cases:

I agree that PB lacks capacity in certain situations, for example because of anxiety, mental disorder or influence. I don’t know if she has capacity in optimal circumstances, but I have not seen sufficient evidence that she lacks capacity then.^78^

But also (and ‘in dynamic interaction’) so do emotional factors. Thus her capacity to weigh information: “… is further impeded by her ambivalence (mixed feelings, ‘confusion’) about her husband and the pressure he seems to place on her to have a family. The latter [pressure] is contributed to (a) by Mrs A’s personal characteristics, associated with both her learning disability and her personality, such as her eagerness to please, her suggestibility and her tendency to acquiescence and (b) by Mr A’s personal characteristics, including a suspicious and hostile stance in relation to support services, leading to his giving Mrs A mixed messages about what is in her interests, thereby ‘confusing her’ more and therefore incapacitating her further.^79^

Some judgments included nuanced discussion of how P’s ‘impairment of mind or brain’ was said to interact with interpersonal influence in a dynamic fashion; for example, through increasing anxiety in the subject, or through a learning disability rendering someone ‘eager to please’ others. Not all of these references explicitly used the term ‘causative nexus’ to describe this.^80^

The case-based analysis which follows seeks to organize the ways judges deal with evidence of influence and impairments in the more complex cases. We first consider two landmark cases, then proceed to examine two contrasting approaches taken by judges since then. To provide sufficient context to each case, we have included some quotes that were not part of the final sample of 54 references.

#### 1. Landmark cases

The term ‘dynamic interaction’ is used in *A Local Authority v Mrs A*,^81^ in which P was a 29-year-old married woman with a learning disability. When providing evidence, the consultant psychiatrist implied that Mrs A’s capacity to take a decision to use contraception was a function of various interpersonal dynamics. The pressure applied from her husband, their joint relationship with services, and her own impairments were all considered part of a ‘dynamic interaction’ that was relevant to her decision-making abilities.^82^ When making his final judgment, Bodey J seemed to accept the evidence that Mrs A had understood all necessary elements required to decide whether to take contraception. He was also ‘satisfied that her decision not to continue taking contraception is not the product of her own free will’.^83^ Nonetheless, Bodey J controversially ruled that Mrs A did not have capacity to take this decision, on the basis that she could not use or weigh due to Mr A’s extreme pressure. The judgment as to whether contraception was in Mrs A’s best interests was then delayed until more meaningful engagement with Mrs A and her husband could take place. It should be noted that the decision (which predated that of the CoA in *PC v NC v City of York Council*)^84^ is, strictly, difficult to align with the MCA 2005 because it was not clear whether Bodey J found that Mrs A lacked capacity to take a decision regarding contraception because of the effect of her learning disability (ie, she lacked capacity applying the MCA 2005), or because of the influence of her husband (which would not satisfy the test under the MCA 2005). We revisit this point in the discussion below.

The issue of dealing with influence was more adequately addressed, in MCA 2005 terms, in *PC v NC v City of York Council*,^85^ and which we discussed in Section IV.B.5 regarding the interpersonal aspect. On appeal, PC successfully contested the original ruling that she did not have capacity, partly on the basis that the previous judge had not adequately spelled out the causative nexus. The appeal judges were cautious to ensure that the causative nexus was not ‘watered down’^86^ by accepting evidence that implied a weaker than causal relationship between the impairment and functional inability. To do so would, in their analysis, have led to an outcome-focused approach, which was incompatible with the MCA 2005. Given that PC was previously found to have capacity to marry NC, and that there was no evidence of any worsening of her learning disability since then, the CoA clearly did not consider itself to have the jurisdiction to infringe on either her decision to marry, or decisions closely related to it.

#### 2. Separating influence and impairment

Since the decision in *PC*,^87^ judges have generally approached the causative nexus by viewing impairment and influence as distinct and separate, and clearly specifying which of the two caused P’s inability. This has been used to settle several disputes between expert witnesses. For example, in *NCC v PB and TB*,^88^ both witnesses referred to both the husband’s (TB’s) influence on PB and her anxiety affecting PB’s ability to make decisions with respect to her care, contact and residence (see the quote in Section IV.D below). While the second opinion psychiatrist had deliberated over whether TB’s influence had rendered PB incapacitous ‘all the time’, he ultimately argued that PB’s capacity was ‘finely balanced’.^89^ Conversely, the treating psychiatrist ‘regarded PB and always has as clearly lacking capacity’.^90^ In her judgment, Parker J sided with the latter evidence, and stayed loyal to the decision in *PC* by regarding ‘PB’s condition as the cause of her inability to use or weigh’.^91^ She held that PB met the threshold for lacking capacity as a result of her mental illness, with TB’s influence contributing to this even when he was not present.

Similarly, in *London Borough of Redbridge v G*,^92^ a dispute took place regarding the role of the impairment and influence in relation to capacity (see Section IV.B.2 above). The psychiatrist argued that both dementia and influence were compromising G’s decision making, whereas the social worker argued that this inability was only caused by the influence and controlling behaviour of G’s carers. Russell J preferred the psychiatrists’ evidence and specified her detailed reasons as to why G’s dementia rendered her incapable of taking the relevant decisions.^93^ Russell J accepted several of the social worker’s arguments on the influencing relationship; however, only the psychiatrist was instructed to give evidence on G’s impairment. In effect, once the influence and impairment had been separated, this meant that only the psychiatrist provided evidence on which to decide whether the MCA 2005 should apply.^94^ Russell J went on to rule that G lacked capacity to take the relevant decisions and was also subject to undue influence by her carers, thus allowing jurisdiction under the MCA 2005.^95^ Although Russell J made a clear case for a causal relationship between G’s impairment and inability, it is noteworthy that the published judgment did not cite *PC*. Given the depth of evidence on both influence and impairment, one might consider Macfarlane LJ’s warnings over ‘watering down’^96^ the causative nexus to be particularly relevant in this case.

Several years later, in *Re B (Capacity: Social Media: Care and Contact)*,^97^ a young woman with a learning disability was considered to be at risk from a third party, Mr C (as discussed in Section IV.B.5 above). During his general observations on the causative nexus, Cobb J remarked, in very clear terms, that:

I am satisfied that influence is a factor, but I share the view of Dr. Rippon that it is not actually operative on her decision making and is in any event not more significant than the clearer evidence about impairment of the mind.^98^

In justifying his focus on the learning disability as separate to influence, he cited the precedent set by Parker J above.^99^ Hence, Cobb J ruled that B could not use or weigh the relevant information to take a decision as to contact with Mr C, due to her learning disability.

#### 3. Mixing influence and impairment

However, some judges explicitly spelled out the difficulty with separating the impairment from the influence. In *Newcastle Upon Tyne City Council v TP*,^100^ the CoP sought to determine whether TP, a woman with a learning disability as well as cerebral palsy, had capacity to take decisions regarding her social care, medical treatment, contact, and finances. TP had wished to live in the community, ideally with her partner (FW), who also had a learning disability. The local authority wished for TP to stay in residential care, and they had previously intervened with men she had befriended due to concerns that she was being financially exploited. During proceedings, the court heard evidence from the clinical psychologist that it was ‘difficult to tease out what is influencing TP’s decision making’.^101^ Moir J also found it difficult to distinguish between the influence and capacity, particularly with regard to contact. Regarding capacity for contact, she remarked, for example, that ‘it is only because of TP’s learning disability that the coercion has been able to take such effective hold. Both exist together’.^102^ The published judgment did not cite any of the influence landmark cases, though Moir J’s reasoning briefly focused on TP’s other relationships and functioning when FW was absent.^103^ Moir J ultimately ruled that the evidence pointed towards TP having lacked capacity to take these decisions because of her learning disability.

In the proceedings reported as *London Borough of Brent v NB*,^104^ Senior Judge Hilder cited *PC*,^105^ and reaffirmed that the court must be satisfied that the impairment itself causes an inability to take a decision, rather than other factors in the person’s life. However, the expert clinical psychologist considered that MB was vulnerable to influence and recommended that family therapy take place as a practicable step to potentially improve MB’s enmeshed maternal relationship and possibly regain his capacity. While ‘practicable steps’ usually target the inability caused by an impairment of mind or brain, here the therapeutic focus was MB’s relationship with his mother. Senior Judge Hilder’s decision to delay proceedings for this relational intervention, suggests the mixing of influence and impairment in the domain of decision-making support. If Senior Judge Hilder had decided to separate the influence and impairment during the initial proceedings, then she might have been satisfied (as she later ruled) that MB’s learning disability and brain injury caused his lack of capacity. Under such an approach, it would have been difficult to see how interventions to improve the influencing relationship could lead to MB regaining capacity, without targeting the impairments themselves.

Following delays, MB received four sessions of family therapy, which did not produce any notable improvement. Interestingly, the family therapist submitted that intervening in enmeshed relationships ‘is not achievable in a few short weeks’.^106^ There was some dispute, from MB’s father’s representative, as to whether MB had been provided with enough support, given that the therapy was unfinished and met with practical problems. Nonetheless, Senior Judge Hilder ruled that she was satisfied that the court had exhausted all practicable steps. She subsequently ruled that MB lacked capacity because of his brain injury and learning disability.^107^

## V. DISCUSSION

### A. Overview of main findings

The role of influence, and when it becomes undue, is one that has exercised the courts and commentators for many years. However, traditionally, the focus has been upon examining the situation in retrospect, where the question is whether a particular action should be viewed as having been taken under the undue influence of another; for instance, whether a will should be accepted as valid, or whether a gift or contract should be set aside. There has been much less focus on how relational matters should be considered in ‘real time’, in circumstances where the question is about what is to be done to secure the interests of the person in question going forward. This is the first systematic content analysis of interpersonal influence problems in cases before the English courts where this issue has been the subject of consideration and determination.

Our search returned 20 published cases for which influence from a named person was cited by the courts as relevant to P’s capacity. We then undertook a qualitative content analysis, during which we identified five main types of interpersonal influences towards P. Influence problems were construed as P having an inability to preserve their free will or independence, as restricting P’s perspective, as valuing or dependence on a relationship, as acting on a general suggestibility to influence, or as P denying facts about the relationship. Most of the statements were used as evidence of the person’s functional abilities and when discussing the causative nexus. Our results challenge perceptions of mental capacity as an individualized construct and show that the courts are attributing importance to relational factors across a range of decision-making situations and conditions.

Out of the four functional abilities, interpersonal factors were most likely to be cited as justification that the person can or cannot ‘use or weigh’ relevant information. Our finding here is unsurprising, given that ‘using or weighing’ is the most contested part of the functional test, the broadest in scope, and overlaps conceptually with ‘understanding’.^108^ This part of the analysis is important insofar as it shows that judges have had to explore how they considered interpersonal influence to be operative on the abilities set out in the MCA 2005, and did so at a granular level, rather than merely considering them in abstract. On this evidence, and perhaps contrary to the position sometimes advanced that the MCA 2005 is unable to take into account interpersonal factors,^109^ the cases show that it is seems to be able to do so, albeit without always a clear mechanism being identified.

Our third category, ‘valuing or dependence on the influencer or the relationship’, suggests that judges may look at the relational context to infer whether P is making an unwise decision, rather than an incapacitous one.^110^ These statements show that interpersonal influence can go either way as a rationale for establishing whether P was unable, or simply unwilling, to use or weigh information relevant to the decision. P might be deemed unable to use or weigh because they are overvaluing a relationship on which they are dependent, as in *Re MRJ*.^111^ In another context, P might be deemed unwilling to use or weigh because of their strong family values, as in *London Borough of Croydon v KR*.^112^ One challenge for the CoP will be to demonstrate that it is able satisfactorily to be able to distinguish between these two scenarios and to justify how they are different.

Although we only included cases with a named influencer, we should note that sometimes the courts widened their analysis to look at whether P was susceptible to problematic influences in a general sense. The statements within our ‘suggestibility’ category indicate that the idea of vulnerability has been used to argue that P lacks mental capacity, even though there is no mention of vulnerability or suggestibility within the MCA 2005. When used in the MCA 2005 context, it seems the courts partly see this vulnerability as inherent to P as opposed to only situational.^113^ This framing has wide ranging implications. In one case, an expert witness explicitly stated that they could have negatively influenced a ‘suggestible’ person if they wanted to, which raises questions as to why influence problems only seem to be litigated when involving families, friends or carers.^114^ The idea of labelling people as ‘suggestible’ is highly sensitive and could easily perceived as stigmatizing towards the person who has been subject to influence, particularly where this shades into abuse, and downplaying or taking responsibility away from those exerting the influence.^115^ And in cases where the court is required to make decisions about P’s best interests, there are clearly implications as to the extent to which the court can or should take into account their wishes and feelings if those might, in fact, reflect the wishes and feelings of those around them.^116^

We were also able to identify how judges handled evidence of a possible interaction between P’s impairment and the interpersonal influence. *A Local Authority v Mrs A* was the first case to do so and referred to this as the ‘dynamic interaction’.^117^ However, this judgment is, with respect, legally incoherent because it did not, in fact, identify which was the material cause of Mrs A’s inability to make the decision in question: was it the impairment, or the influence of her husband, or some combination of the two?^118^ The next case in our sample to address this, *PC v NC v City of York Council*,^119^ stressed that there must be a causal relationship between the impairment and the functional inability to find P as lacking capacity. Since then, judges have appeared to handle evidence of influence cautiously with respect to the causative nexus.

Interestingly, these legal tensions have also manifested in Singapore, where in effect the same legislation is applied in a far more relational manner. For example, in *Re BKR*,^120^ the Singapore CoA came to the conclusion that the wording in section 1(4) of the MCA 2005 in Singapore:

do[es] not suggest that there can be no other cause of P’s inability to make decisions besides mental impairment; we do not think that those words indicate that the MCA was intended to exclude situations in which the inability to decide was caused by both mental impairment and P’s actual circumstances.^121^

Instead, the CoA found her to lack capacity on the basis of *both* her mental impairment *and* the circumstances in which she lived, in circumstances where the Court accepted that it was unrealistic to imagine that BKR could simply be removed from those circumstances. The approach here seems to contrast to that which most judges explicitly take in the CoP, though, in our sample, most cases with similar disputes still found the person to lack capacity (see Section IV.D above).

Nonetheless, we have found several examples in which the expert witness reasoned that the influence and the impairment had a multiplicative impact on the P’s functional abilities. It is also clear that several experts believe that extreme social pressure can have a substantial impact on a person’s decision-making ability in its own right. Judges are then tasked, on one view, with separating the influence from the impairment. This raises questions as to whether judges are well equipped to distinguish between these two factors, and whether the conclusions they reach are either overly inclusive (ie, ruling that a person lacks capacity to take a decision when, in reality, they are ‘merely’ under extreme pressure),^122^ or under-inclusive (failing to identify that, taken together, the influence and the person’s impairment means that they cannot, in fact, make the decision in question for purposes of the MCA 2005). There are risks with either approach, but perhaps the greatest risk is that those concerned (from the judge downwards) are not clear about the basis upon which they are proceeding.

Finally, the cases also shed light on how the criteria for certain decisions require some basic ability to engage with social information. For example, the information relevant to making decisions about care arrangements includes knowing that carers might not always provide the necessary support and that one can complain if this happens. Similarly, the legal test for making decisions about contact requires some ability to judge the positive and negative aspects of having contact with that particular person.^123^ Arguably, such tests present an opportunity for the courts to consider relational factors within a capacity context. Also, if P makes an unwise decision, it can prove difficult to distinguish whether P’s apparent lack of engagement with social information is caused by their impairment, as opposed to their values or ambivalence. One way to introduce greater clarity and consistency may be to consider the psychological concept of theory of mind; the cognitive ability to consider the intentions, motivations, likely behaviours and perhaps even the competence of others.^124^ The concept of theory of mind has been researched in people with various ‘impairments’, most notably autism.^125^ Further investigation may help to identify those who have been *unable* to process social information due to an impairment, as well as those who are able to, but might be considered at present to be disabled from doing so by third party influence. Both groups may be helped to have capacity through tailored practical supportive steps.

### B. Implications for clinical practice

In this article we have highlighted several practical and policy considerations for professionals who assess capacity and added nuance to the concept of an ‘optimal’ capacity assessment. Our analysis shows that professionals are perceiving third party influence to be a problem, particularly for social care service users. A previous study has shown that many professionals tasked with capacity assessment are concerned about undue influence,^126^ but it has been less clear how these concerns are dealt with. Some professionals appear to be struggling to resolve these concerns within their local safeguarding team and may therefore seek a legal solution by bringing these cases to court.^127^ Leading up to this, professionals have often made unsuccessful attempts to engage with P and/or their family, friends, and carers, and they often have some suspicion that P is potentially at risk or vulnerable to exploitation, harm, abuse or neglect, either from that specific person or in general.^128^ By proceeding with litigation, professionals may have sought to avoid making a determination which carries only risk for them; either of being seen to be grossly infringing on P’s private life or allowing them to remain in a dangerous situation^129^ In any case, we have demonstrated that the courts are willing to scrutinize these concerns, both in terms of the MCA 2005 and the inherent jurisdiction.

One key consideration from our study is that problematic influences have been constructed by the courts as interacting with and temporarily affecting P’s functional abilities. We argue that, in accordance with the MCA 2005’s principle of taking practicable steps to support P’s decision making,^130^ the capacity assessment process should continue for as long as required for potentially problematic influences to be assessed. If appropriate, these should be mitigated during the assessment so as to support P to meet the requirements of each specific decision in question.^131^ Capacity is, of course, time-specific and so it may be necessary to reach a capacity determination before this point. In this case, appropriate measures to provide support (either on the basis of informed consent or best interests, depending on the capacity outcome) might still be continued and these might form practicable steps down the line. As such, the determination should not be taken as holding indefinitely, but rather pending further—identified—work. Even in one case where P was determined to lack capacity, and best interests proceedings were initiated, the judge recommended that a repeat capacity assessment take place in the near future.^132^

In half of the cases in our study, P was diagnosed with an intellectual disability. This is a slightly higher proportion than in a sample of 170 personal welfare cases in the CoP (41.18%),^133^ and higher than in another sample of published disputed capacity cases (37.50%).^134^ This is consistent with evidence from a recent survey, which suggested that professionals are most concerned about undue influence when assessing capacity in people with intellectual disabilities, as well as people with dementia and older adults.^135^ Professionals who work with these populations might thus particularly benefit from conceptualizing the different types of influence considered by the courts.

Professional guidance on interpersonal factors during capacity assessments is largely scarce, with a few notable recent exceptions. The National Institute for Health and Care Excellence (NICE) guideline on decision-making capacity, for example, mentions that professionals should be aware of undue influence and coercion.^136^ The British Psychological Society have issued guidance for clinical psychologists, which made brief references to ‘social influence’ during capacity assessments.^137^ Although this is a positive shift, the mentions in both documents are brief and made with little to no supporting evidence, from either the case law or empirical research. The Social Care Institute of Excellence have also published a chapter advising social workers that both the MCA 2005 and inherent jurisdiction are available to safeguard adults.^138^ This does not, however, provide any additional guidance on how to reach the capacity determination. Developments such as the revision of the MCA 2005 Code of Practice, albeit somewhat stalled at the time of writing, may present some opportunities to promote a better understanding towards the nuances of the relational context.^139^

### C. How should the courts handle relational issues?

Our typology provides clear evidence that the courts are developing an approach to the policy gap outlined by the Law Commission in 1993, which identified that some vulnerable adults would lie outside the scope of the MCA 2005 and other frameworks.^140^ The MCA 2005 did not explicitly address these concerns, and some scholars have argued that a reformed MCA 2005 would provide for interpersonal issues, which causally affect the person’s ability to consent, to be considered within a capacity framework.^141^ There is, at present, no immediate prospect of reform of the MCA 2005, but our study has shown that the courts are not waiting for reform and, instead, are grappling with these matters live. So far, there seem to have been two trends in the courts’ approach. First, judges may use an expansive approach to the causative nexus to infer that P does not have capacity when under the deleterious influence of another person. As we have demonstrated in Section IV.B. above, several experts have made the case that when an impairment of mind or brain *and* an external influence are both present, they may act in tandem to worsen a person’s decision-making ability. This suggests that expert witnesses do not interpret the causative nexus as meaning that interpersonal problems are irrelevant to capacity determinations. On hearing this evidence, several judges have also found it difficult (or impossible) to separate the impairment from the influence. This would suggest that the Law Commission’s description of the functional test as a ‘cognitive test’ is being broadened and that their vague term ‘true choice’ is starting to gain greater specificity as influence cases get analysed in court.^142^ An expansive approach could encourage judges against separating the impairment from influence in cases where such distinctions would prove arbitrary and decontextualizing. At present, the MCA 2005 does not expressly mention interpersonal factors, although as is evident in the case of Singapore (discussed in Section V.A. above) it does not prohibit their consideration.

Secondly, judges may decide that to reach a determination of incapacity on the (partial) basis of interpersonal factors is either morally or legally unjustified. If they then find that the person has capacity, the only way to safeguard their interests is to use the inherent jurisdiction of the High Court. The bounds of the inherent jurisdiction are loosely defined, and the precise scope of what is, or is not, permissible when it comes to protection is unclear. To some, this may appear advantageous as it allows for protection beyond the MCA 2005 in exceptional circumstances, on a case-by-case basis.^143^ To others, especially those who perceive the MCA 2005 as safeguarding autonomy, it may appear discomforting that such a framework can exist, untrammelled by statutory limits, and which can go against the wishes of people who have capacity.^144^ It is also important to note that the inherent jurisdiction lacks the principled approach of the MCA 2005, including its straightforward provisions to provide support, as both practicable steps to maximize capacity and via a best interests process predicated on returning the person to autonomy. Indeed, some may argue that such extraneous factors might guide judges toward the MCA 2005 as a more workable framework.^145^

Ultimately, it may be thought that this is a question which can and should only be resolved by Parliament, because it represents a societal determination of how broadly the net of protection should be cast, and how fine-grained that net should be. In the meantime, some predict that more hard cases will test the limits of the MCA 2005,^146^ which would likely include borderline capacity cases in which there is suspicion of coercion or undue influence. Perhaps the most important message from our study is the continued need for transparency during capacity assessments. It seems that the more granular the analysis, the more defensible the narrative as to whether P really lacks capacity under the MCA 2005, or is a capacitous, but vulnerable, adult.^147^

### D. Future directions

Future studies should explore the various ways in which interpersonal influence can be constructed using a larger sample of capacity assessments. Different methods may shed light on topics that are unlikely to arise in published court judgments, such as how interpersonal influence from health and social care professionals (as opposed to the ‘usual suspects’ of family members) is handled outside of litigation. Future studies should also consider interpersonal issues specific to different clinical populations, how to support people subjected to potentially problematic influences, and how these models of influence may vary between different cultures. Examples of study designs may include semi-structured interviews with persons whose capacity is being assessed or expert witnesses, surveys or case-based vignettes with professionals, or analyses of expert evidence submissions. The demographic data collection should be detailed enough to identify priority areas for these research interests.

The work that has already started to conceptualize the place of relational influence within the MCA 2005 should be continued and tested.^148^ Beyond legal analysis, it will also be critical to further explore the mechanisms by which interpersonal factors might be said to interact with various impairments to affect decision making; that is how influence operates on impairment and vice versa. Psychology and cognitive neuroscience studies can better elucidate these mechanisms and guide development of support. Finally, future studies will no doubt want to explore the extent to which the COVID-19 pandemic changed matters in relation to specific examples of interpersonal influence. While protection under the MCA 2005 requires that the person has an identified mental impairment, the recent and unprecedented challenges have made it clear that anyone (including those with only physical health problems)^149^ may become vulnerable at any point.

We also note that our work throws further light on a pressing debate. Human rights scholars have increasingly advocated for models of ‘supported decision making’ to replace ‘substituted decision making’ on the behalf of adults with impairments. This movement, rightly, seeks to implement policies that, insofar as possible, recognize a person’s legal capacity—their right to make decisions for themselves. However, support can easily shade into deleterious influence, and vice versa. This highlights the need for conceptual and societal clarity as to the markers for the points where that influence goes beyond acceptable limits. For example, those who require substantial support are generally more dependent on those providing it, which is a risk factor for undue influence.^150^ Indeed, Article 12(4) of the United Nations Convention of the Rights of Persons with Disabilities (CRPD) states that ‘measures relating to the exercise of legal capacity respect the rights, will and preferences of the person [and] are free of conflict of interest and undue influence’^151^ One challenge raised by our study is how to identify the line between support and undue influence.

### E. Limitations

It will be noted that the search period pre-dated the coronavirus pandemic. This was not by design; the impact of pandemic-related demands upon the study team meant that it has not been possible to carry out a further review to bring the search closer to the present day. However, given the short gap between the end of the review period in November 2019 and the start of the pandemic in March 2020, the review serves to indicate how the courts were operating with the concepts of capacity and vulnerability before the additional extraneous challenges of the pandemic took effect.

Given the complexity of the data, there were limitations to testing the reliability of the coding schemes. At a conceptual level, the themes were not completely distinct, so we decided to use relatively large blocks of text to preserve the full context in which the statement was made. As a result of our approach, we could not calculate a standard kappa statistic, which requires mutually exclusive categories.^152^ Instead, we calculated intercoder agreement using an extension of the kappa statistic, which has been adjusted for holistic coding.^153^ We argue that this is approach is more applicable for an ecologically valid coding scheme, and that the alternative would have overly condensed the references or the coding scheme and thereby risked oversimplifying the data.

We only included published judgments from England and Wales within our sampling frame. Only a small number of CoP cases are selected by judges for publication, and it is not logistically possible to estimate the total number of unpublished capacity dispute cases over this period.^154^ The content of each published judgment is also selected by the judge and the level of detail varies. Consequently, our results will reflect the more complex capacity cases likely to result in published judgments, and may not be representative, or exhaustive, of all the ways in which interpersonal influence problems have been construed to impact upon capacity, inside or outside the courts. For example, the majority of cases in the CoP are brought forward by professionals, so perhaps a reason why the courts do not construct professionals as being problematic influences is not because this does not take place, but rather that the courts have not provided an accessible route when P has such claims.^155^ It is also difficult to escape the impression that cases involving the inherent jurisdiction are particularly driven by concerns as to the outcome of the decision(s) in issue since, in effect, its primary use in this context is to prevent a person from making a capacitous but unwise decision.

While the authors of previous capacity case reviews have acknowledged concerns about bias, many have argued that dispute cases are broadly representative of unpublished case files.^156^ Published judgments are especially informative as they tend to be selected based on precedent value to judges and normative value to practitioners.^157^ Perhaps, one of the main strengths of our research is that it raises awareness of these complex issues around capacity and influence, as the courts consider them, and it provides a novel framework to assist with interpreting this. Arguably, a detailed consideration of interpersonal problems could be most useful where the line is most blurred between capacity and vulnerability. Some authors have demonstrated how such reviews lend themselves well to integrative methods, such as in-person observations, interviews and grey literature reviews, to produce complementary findings.^158^ The depth of discussion within these cases should, therefore, provide a strong initial basis from which to evaluate the courts’ approach to dealing with concerns of problematic influences.

## VI. CONCLUSION

There is no detailed guidance to suggest how professionals in England and Wales should approach the question of interpersonal influence when assessing capacity. We have undertaken a content analysis to show that interpersonal influence problems are relevant to capacity judgments across a broad range of decisions and impairments. Our typology provides an early framework to begin conceptualizing the different ways in which interpersonal influence problems can be constructed by the courts. This has the potential to improve clarity around the most contested criteria within the functional model, and to better equip the courts to consider possible interactions between mental impairments and influence. Future research should supplement these findings by providing insights across different clinical groups and ethical positions.

## Ethics

All data are in the public domain and therefore we did not require ethical approval. Data files are available on reasonable request to the corresponding author.

## Footnotes

1 T Grisso and PS Appelbaum, *Assessing Competence to Consent to Treatment: A Guide for Physicians and Other Health Professionals*’ (OUP 1998) 131.

2 T Grisso and PS Applebaum, ‘Thinking about Competence’ (2003) 4 Crime: Critical Concepts in Sociology 25.

3 Mental Capacity Act 2005, s 3. Also, note that with the exception of the communication criteria, these reflect criteria originally endorsed at common law by the High Court, at that point exercising its inherent jurisdiction in *Re C (Refusal of Medical Treatment)* [1994] 1 WLR 290. For a review of the history of the MCA 2005, see A Ruck Keene and others ‘Taking Capacity Seriously? Ten Years of Mental Capacity Disputes before England’s Court of Protection’ (2019) 62 International Journal of Law and Psychiatry 56.

4 See *PC v NC v City of York Council* [2013] EWCA Civ 478 and, more recently (after the period covered by our review), in the Supreme Court judgment *Re JB* [2021] UKSC 52, [78].

5 ibid [58].

6 ibid.

7 Law Commission, *Mentally Incapacitated and Other Vulnerable Adults: Public Law Protection* (Law Comm No 130, 1993) <https://www.lawcom.gov.uk/document/mentally-incapacitated-and-other-vulnerable-adults-public-law-protection/> accessed 29 April 2022, paras 2.18–2.19.

8 C Kong, ‘*Mental Capacity in Relationship: Decision-making, Dialogue, and Autonomy*’ (CUP 2017) 3.

9 For a review of cogntive research on social influence, see W Wood and others, ‘Minority Influence: A Meta-analytic Review of Social Influence Processes’ (1994) 115 Psychological Bulletin 323.

10 See eg, U Toelch and RJ Dolan, *‘*Informational and Normative Influences in Conformity from a Neurocomputational Perspective’ (2015) 19 Trends in Cognitive Sciences 579; E van der Plas and others, ‘Advice-taking as a Bridge between Decision Neuroscience and Mental Capacity’ (2019) 67 International Journal of Law and Psychiatry e101504.

11 See the Safeguarding Vulnerable Groups Act 2006, s 59, for the statutory definition of a vulnerable adult.

12 For similar critiques of whether relational issues should be framed as a ‘mental capacity’ issue, see eg, B Clough ‘What about Us? A Case for Legal Recognition of Interdependence in Informal Care Relationships’ (2015) 36 Journal of Social Welfare and Family Law 128; L Series ‘Relationships, Autonomy and Legal Capacity: Mental Capacity and Support Paradigms’ (2015) 40 International Journal of Law and Psychiatry 81.

13 Law Commission (n 7), para 1.6.

14 See *Re F (Mental Patient: Sterilisation)* [1990] 2 AC 1, 13.

15 See *Re SA (Vulnerable adult with capacity: marriage)* [2005] EWHC 2942 (Fam). The fact that the jurisdiction could be used in relation to both categories of individual—or even the same individual at different points—is illustrated by *Re G* [2004] EWHC 2222. It also meant that there may not have been the same analytic clarity as to which category the person fell into. It is difficult, for instance, to be clear whether Ms T, in *Re T* [1992] EWCA Civ 18, would be seen now as having or lacking the capacity to decide to accept or refuse the blood transfusion she required, as it was not necessary for the courts to disentangle the relative influence of the impact of the accident she had been in and the influence of her mother.

16 In addition to the above, see D Lock ‘Decision-making, Mental Capacity and Undue Influence’ (2015) 20 Judicial Review 1 for a more detailed overview of the pre- and post-MCA 2005 case law around the undue influence and the inherent jurisdiction.

17 See *DL v A Local Authority & Ors* [2012] EWCA Civ 253.

18 It is beyond the scope of this article to provide a full summary of research on the high court’s inherent jurisdiction, but some relevant critiques of its use in undue influence cases include: MC Dunn and others, ‘To Empower or to Protect? Constructing the ‘Vulnerable Adult’ in English Law and Public Policy’ (2008) 28 Legal Studies 234; Ruck Keene and others (n 3); J Lewis, ‘Safeguarding Vulnerable Autonomy? Situational Vulnerability, the Inherent Jurisdiction, and Insights from Feminist Philosophy’ (2021) 29 Medical Law Review 306; P Skowron, ‘The Relationship between Autonomy and Adult Mental Capacity in the Law of England and Wales’ (2018) 27 Medical Law Review 32; J Craigie, ‘Conceptualising ‘Undue Influence’ in Decision-making Support for People with Mental Disabilities’ (2021) 29 Medical Law Review 48.

19 This has motivated the development of practical guidance, including: 39 Essex Chambers, ‘Using the Inhererent Jurisdiction in relation to adults’ (Guidance Notes, 9 November 2020). <https://www.39essex.com/mental-capacity-guidance-note-inherent-jurisdiction/> accessed 29 April 2022.

20 Although the recommendation was taken up in other jurisdictions, see, for instance, the Vulnerable Adults Act 2018 passed in Singapore, a jurisdiction which also has a Mental Capacity Act directly modelled on the MCA 2005.

21 There are numerous examples of research focusing explicitly on supporting capacity, including P Webb and others ‘Key Components of Supporting and Assessing Decision Making Ability’ (2020) 72 International Journal of Law and Psychiatry e101613; N Stanley and J Manthorpe, ‘Small Acts of Care: Exploring the Potential Impact of the Mental Capacity Act 2005 on Day-to-day Support’ (2009) 8 Social Policy and Society 37; Series (n 12).

22 See eg, *Evans & Ors v Lloyd & Anor* [2013] EWHC 1725.

23 Ruck Keene and others (n 3).

24 Examples of cases thereby excluded are *The London Borough of Wandsworth v M & Ors* [2017] EWHC 2435 (Fam) and *London Borough of Southwark v NP & Ors* [2019] EWCOP 48, both decisions relating to 17-year-olds.

25 For a step-by-step overview of content analysis, see M Schreier, *Qualitative Content Analysis in Practice* (Sage 2012) 41. For a more recent overview, including software and research examples, see P Mayring, *Qualitative Content Analysis: A Step-by-Step Guide* (Sage 2021).

26 For a discussion regarding the benefits of content analysis for legal scholarship, see both RF Wright and MA Hall, *‘*Systematic Content Analysis of Judicial Opinions’ (2008) 96 California Law Review 63; N Kane and others ‘Applying Decision-making Capacity Criteria in Practice: A Content Analysis of Court Judgments’ (2021) 16 Plos One e0246521

27 AP Kirilenko and S Stepchenkova, ‘Inter-coder Agreement in One-to-many Classification: Fuzzy Kappa’ (2016) 11 PloS One e0149787.

28 MCA 2005, ss 2 and 3.

29
*PC v NC* (n 4).

30 An example of a ‘mild’ learning disability case is *London Borough of Hackney v SJF & Anor* [2019] EWCOP 8, [6].

31 An example of a ‘moderate’ learning disability case is *A Local Authority v Mrs A and Mr A* [2010] EWHC 1549 (Fam), [7].

32 In one case, P was considered to have been a vulnerable adult because of their unspecified mental health problem (*Redcar & Cleveland Borough Council v PR (No 2)* [2019] EWHC 2305 (Fam)) and in the other, because of their physical health problems (*Southend-On-Sea Borough Council v Meyers* [2019] EWHC 399 (Fam)).

33 Note that the remaining 12 references (from nine cases) did not point to a specific mechanism that was clearly classifiable.

34
*Derbyshire County Council v AC* [2014] EWCOP 38, [44], *per* Cobb J.

35 ibid.

36 ibid [8].

37 A term which broadly refers to complex mental processes that are necessary for goal-directed behaviour, such as attention, working memory, organization, and problem solving.

38 ibid [17].

39 ibid [44].

40
*Southend-On-Sea Borough Council v Meyers* (n 32).

41 ibid [38].

42 ibid [40].

43 ibid [41].

44
*PCT v P, AH, & A Local Authority* [2009] COPLR Con Vol 956, [37] *per* Hedley J.

45 ibid.

46 ibid [37].

47 ibid.

48
*London Borough of Redbridge v G & Ors* [2014] EWCOP 485.

49 See also ‘valuing or being dependent on the relationship’ below.

50
*PCT v P* (46), [54], [79], *per* Russell J.

51 Series (n 12) argued that *London Borough of Redbridge v G* (n 48) was ‘as evidence that the courts treat mental capacity as being partially constituted by freedom from oppressive relationships’. She regarded this aim as a source of instability for judges, as it does not neatly fit into the idea of individual rationality. See Section IV.D.2, where we elaborate on how the courts constructed influence when referring to the causative nexus in our sample.

52
*London Borough of Croydon v KR & Anor* [2019] EWHC 2498 (Fam), [26], *per* KR.

53
*Re MRJ (Reconsideration of Order)* [2014] EWCOP B15, [46] per Lush J.

54
*London Borough of Croydon v KR & Anor* (n 52).

55 ibid [26].

56 ibid [60], referring to the cases in which the High Court’s inherent jurisdiction was developed and confirmed.

57
*London Borough of Hackney v SJF & Anor* [2019] EWCOP 8.

58 ibid [60].

59
*Re MRJ (Reconsideration of Order)* [2014] EWCOP B15.

60 ibid [46].

61
*A Local Authority v WMA* [2013] EWHC 2580 (COP).

62 ibid [4].

63 Consultant Psychiatrist; *Re A* [2016] EWCOP 3, para 20.

64 This framing has potentially wide-reaching implications around the idea of inherent vulnerability, which we briefly discuss in Section IV.A.

65 ibid.

66 ibid [20].

67
*London Borough of Brent v NB* [2017] EWCOP 34.

68 ibid [51], [70]–[75], as examples.

69 ibid [72]. See Section IV.D.3 below, For a discussion on the causative nexus in relation to MB.

70
*B v A Local Authority* [2019] EWCA Civ 913, [33], *per* Cobb J.

71 ibid; *Re B (Capacity: Social Media: Care and Contact)* [2019] EWCOP 3.

72 As per *LBX v K, L and M* [2013] EWHC 3230 (Fam), [45] *per* Theis J.

73 See n 71, [25].

74 We elaborate on the degree to which judges have been loyal to the causative nexus in Section IV.D below.

75
*PC v NC* (n 4).

76
*PC v NC* (n 4), [7].

77
*PC v NC* (n 4), [8]. Quoted from *CYC v PC and NC* [2012] MHLO 103 (COP), [12].

78
*NCC v PB and TB* [2014] EWCOP 14, [67], *per* Consultant Psychiatrist.

79
*A Local Authority v Mrs A and Mr A* (n 31) *per* Bodey J quoting Dr K.

80 Note that the term ‘causative nexus’ was only developed by the CoA in 2013 in *PC v NC* (n 4).

81 See n 31.

82 ibid [51].

83 ibid [73].

84
*PC v NC* (n 4).

85
*PC v NC* (n 4).

86
*PC v NC* (n 4), [58].

87
*PC v NC* (n 4). Also see Lock (n 16) for an overview of these landmark cases, as well as pre-MCA 2005 case law.

88 See n 79.

89 ibid [63], [67], [69].

90 ibid [69].

91 ibid [92].

92 (n 48).

93 ibid [76]–[81].

94 Authors of other CoP reviews, such as Mary Donnelly, Paula Case and Jaime Lindsey, have expressed concerns over court deference to evidence from medical professionals over other professions: See M Donnelly, ‘Capacity Assessment under the Mental Capacity Act 2005: Delivering on the Functional Approach?’ (2009) 29 Legal Studies 464; P Case, ‘Negotiating the Domain of Mental Capacity: Clinical Judgement or Judicial Diagnosis?’ (2016) 16 Medical Law International 174; J Lindsey, ‘Competing Professional Knowledge Claims about Mental Capacity in the Court of Protection’ (2010) 28 Medical Law Review 1. Lindsey, for example, has argued that there is an implicit ‘hierarchy of professional evidence’ in capacity cases, favouring the perceived ‘objective’ expertise of psychiatrists over the ‘experiential’ social workers. In our analysis, G’s case highlights how such perceptions of expertise may affect which constructions of interpersonal influence are accepted by the courts.

95 In Section V.C below, we elaborate on the boundary issues between the MCA 2005 and the high court’s inherent jurisdiction in the context of capacity and relationships.

96
*PC v NC* (n 4), [58].

97 See n 72.

98 ibid [19].

99 See n 79.

100
*Newcastle Upon Tyne City Council v TP* [2016] EWCOP B4.

101 ibid [32].

102 ibid [37].

103 See relevant references to ‘suggestibility’ in Section IV.B.4 above.

104
*PC v NC* (n 4), [58]. See Section IV.B.5. above for the background.

105
*PC v NC* (n 4).

106 ibid [75].

107 Craigie (n 18), [70] cited the rulings in both *London Borough of Redbridge v G* (n 48) and *London Borough of Brent v NB* (n 68) as evidence of ‘fundamental ambivalence’ in the law, regarding how ‘the influence of others can impact on mental capacity’. One noteworthy difference is that, in the latter case, the psychologist’s evidence on influence delayed the judge’s decision on the causative nexus, whereas the social worker’s evidence did not. Perhaps, this indicates some desire for a more pragmatic approach to dealing with influence in a capacity context.

108 See n 3.

109 For an overview of the literature, including these arguments, see Series (n 12).

110 Note that a person cannot be found to lack capacity solely on the basis of an unwise decision under s 1(4) of the MCA 2005 (n 3).

111 See n 49.

112
*London Borough of Redbridge v G* (n 48).

113 Dunn and others (n 18), offer an especially relevant analysis of how accounts of situational and inherent vulnerability have been used to justify interventions under the inherent jurisdiction, in cases shortly before the implementation of the MCA 2005. In their analysis of both *Re SA* and *Re G*, the court constructed P’s inherent vulnerability as automatically increasing the risk of circumstances in which P would be subject to malign intentions or influence. They concluded that narratives of inherent vulnerability risk ‘reawakening the ghost’ of a status-based approach to capacity [244].

114
*Re A* (n 63), [20].

115 Lindsey’s review of CoP judgments and case files also found several references to P’s inherent vulnerability. She argued that such narratives contribute to a culture in which P is often excluded or limited from participating during proceedings: J Lindsey, ‘Testimonial Injustice and Vulnerability: A Qualitative Analysis of Participation in the Court of Protection’ (2018) 28 Social & Legal Studies 4.

116 Some cases focused on best interests may also be of note, as these were not in scope for the present study. For example, in *ADS v DSM* [2017] EWCOP 8, [37], Charles J was critical of the best interests process which had not, in his view, given ‘consideration’ to the ‘possibility’ that P was ‘vulnerable to influence’ from others, or how influence might need to be ‘minimised’ to obtain P’s authentic will and preference.

117 See n 31, [51], *per* Bodey J.

118 This might have been because this case was heard relatively early in the life of the CoP, at a time when at least some judges might still have been more familiar with the position pre-MCA 2005 when, as discussed in Section IV.D.1, they would not have had to identify which was the operative mechanism in determining whether to grant relief under the inherent jurisdiction.

119 See n 4.

120
*Re BKR* [2015] SGCA 26.

121 ibid [87].

122 Donnelly (n 94) 145 has argued that treating undue influence as a mental capacity issue risks leading the courts to find a person to lack capacity simply based on the behaviour of third parties, thereby shifting the focus away from the real cause of the problem that could potentially be alleviated.

123 Both of these tests, for care arrangement and contact decisions, were specified by Theis J in *LBX v K & Ors* (n 72).

124 See eg, CD Frith and U Frith, ‘Mechanisms of Social Cognition’ (2012) 63 Annual Review of Psychology 287; U Frith and CD Frith ‘Development and Neurophysiology of Mentalizing’ (2003) 358 Philosophical Transactions of the Royal Society of London Series B: Biological Sciences 459.

125 I Leudar and others, ‘Theory of Mind: A Critical Assessment’ (2004) 14 Theory & Psychology 571.

126 As shown in Section IV.D.3 above, *London Borough of Brent v NB* (n 68) provides a useful example of a case in which a judge regarded mitigating influence as a practicable step to supporting capacity. See *London Borough of Hackney v SJF* (n 30) [19] for another example.

127 As Lock notes (n 16), the role of P in influence cases has changed over time. Many pre-MCA 2005 cases invoking the inherent jurisdiction were brought forward by P, seeking to overturn their earlier decisions, rather than by third parties. More recently, public bodies have sought such orders to overturn P’s capacitous decisions, while P may actively defend their initial decision. This could be attributed, in part, to the broad criteria outlined in *Re SA* (n 15).

128 This echoes a point made, by Margaret Isabel Hall, that a capacity assessment might be triggered by concerns about vulnerability, as opposed to only capacity per se: M Hall, ‘Mental Capacity in the (Civil) Law: Capacity, Autonomy, and Vulnerability’ (2012) 58 McGill Law Journal 61, 70.

129 For example, Dunn and others (n 18) have argued that recent abuse scandals in intellectual disability services have strengthened the drive, from health and social care services, to protect vulnerable adults using the inherent jurisdiction. Lock (n 16) has also speculated that local authorities could face more damages claims for failing to use such discretionary powers to protect vulnerable adults from abuse, as similar to cases of children with abusive parents.

130 Mental Capacity Act 2005 (n 3), s 1(3).

131 See n 126.

132 See *Derbyshire County Council v AC* (n 34), [.

133 L Series and others, *Welfare Cases in the Court of Protection: A Statistical Overview* (September 2017) <https://orca.cardiff.ac.uk/118054/1/Series%20Fennell%20Doughty%202017%20Statistical%20overview%20of%20CoP.pdf> 43, accessed 29 April 2022.

134 See n 3.

135 K Ariyo and others, ‘Experiences of Assessing Mental Capacity in England and Wales: A Large-scale Survey of Professionals’ (2021) 6 Wellcome Open Research 144.

136 National Institute for Health and Care Excellence (NICE), ‘Decision-making and Mental Capacity’ (NICE, 2018) <https://www.nice.org.uk/guidance/ng108> paras 1.2.1, 1.2.4, 1.2.11, and 1.4, accessed 29 April 2022.

137 British Psychological Society (BPS), ‘What Makes a Good Assessment of Capacity*?* (BPS, 2019) <https://www.bps.org.uk/sites/www.bps.org.uk/files/Policy/Policy%20-%20Files/What%20makes%20a%20good%20assessment%20of%20capacity.pdf> 30, accessed 29 April 2022.

138 Social Care Institute for Excellence (SCIE), ‘Gaining Access to an Adult Suspected to Be at Risk of Neglect or Abuse: A Guide for Social Workers and their Managers in England’ (SCIE, 2014) <https://www.scie.org.uk/safeguarding/adults/practice/gaining-access> accessed 29 April 2022.

139 Note that the latest draft of the revised MCA 2005 Code of Practice advises professionals to go down the capacity route as long as the impairment can be demonstrated as ‘a cause’ of the inability, and uses the more specific term ‘real reason’ for influence. Compared to the status quo, this might be argued to more firmly discourage use of the inherent jurisdiction until the capacity question is exhausted—why? See Office of the Public Guardian, ‘*Mental Capacity Act 2005 Code of Practice Including the Liberty Protection Safeguards* [draft]’ (Department for Health and Social Care 2022) <https://assets.publishing.service.gov.uk/government/uploads/system/uploads/attachment_data/file/1061499/draft-code-of-practice.pdf> paras 4.48–4.50, accessed 29 April 2022.

140 Law Commission (n 7), paras 2.18–2.19.

141 C Kong and A Ruck Keene, *Overcoming Challenges in the Mental Capacity Act 2005: Practical Guidance for Working with Complex Issues* (Jessica Kingsley Publishers 2018) 140.

142 Law Commission (n 7), para 2.19.

143 Arguments in favour of this ‘safety net’ for vulnerable adults include those advanced by McFarlane LJ in *DL v A Local Authority* (n 17), [53], as well as those made by Clough (n 12) and J Herring and J Wall, ‘Autonomy, Capacity and Vulnerable Adults: Filling the Gaps in the Mental Capacity Act’ (2015) 35 Legal Studies 698. Laura Pritchard-Jones has also tentatively argued that the inherent jurisdiction might be better suited than capacity law to promote autonomy in older adults, if implemented alongside effective advocacy and supported decision-making systems: L Pritchard-Jones ‘Ageism and Autonomy in Health Care: Explorations Through a Relational Lens’ (2017) 25 Health Care Analysis 73, 85.

144 For example, Dunn and others (n 18), [253] have argued that such measures ‘will not provide the adequate procedural and conceptual safeguards to guarantee that “vulnerable adults” are empowered, when protective interventions are deemed necessary’.

145 Similar arguments have been made in A Ruck Keene ‘The Inherent Jurisdiction: Where are We Now?’ (2013) Elder Law Journal 88. This also relates to Lindsey’s (n 95) conclusion that capacity law is used to deal with abuse concerns because of weaknesses in legal adult safeguarding frameworks, though this was not the focus of her paper.

146 N Kane and others, ‘Avoiding Hard Capacity Assessments Will Not Help’ (2020) 216 British Journal of Psychiatry 165.

147 Catriona Mackenzie raised the importance of finer grained analyses to distinguish between different sources of vulnerability, which makes it easier to separate between avoidable and unavoidable vulnerability: C Mackenzie ‘The Importance of Relational Autonomy and Capabilities for an Ethics of Vulnerability’ in C Mackenzie and others (eds), *New Essays in Ethics and Feminist Philosophy* (OUP 2014). Jill Craigie has also concluded, in a similar review (n 18), that fine-grained analyses are needed to decide how appropriate such constructions of influence are to the context in which they are used.

148 See eg, C Kong and A Ruck Keene (n 141), the ‘modes of influence’ by Craigie (ibid) and the approaches to supporting legal capacity outlined by L Series (n 12).

149 As was the case in *Southend-On-Sea Borough Council v Meyers* (n 32).

150 The boundaries between support and influence have been the focus of several articles, including J Craigie, ‘A Fine Balance: Reconsidering Patient Autonomy in Light of the UN Convention on the Rights of Persons with Disabilities’ (2015) 29 Bioethics 398; MJ Quinn ‘Undue Influence and Elder Abuse: Recognition and Intervention Strategies’ (2002) 23 Geriatric Nursing 11; S Wood and PJ Liu ‘Undue Influence and Financial Capacity: A Clinical Perspective’ (2012) 36 Generations 53.

151 See also Committee on the Rights of Persons with Disabilities, General Comment No 1, art 12: Equal recognition before the law (United Nations, 2014), para 37.

152 See US General Accounting Office, *Content Analysis: A Methodology for Structuring and Analyzing Written Material* (General Accounting Office 1996) 20; JR Landis and GG Koch ‘An Application of Hierarchical Kappa-type Statistics in the Assessment of Majority Agreement among Multiple Observers’ (1977) 33 Biometrics 363.

153 See n 27.

154 As Series and others have noted, there have been several methodological obstacles to obtaining detailed and representative data from unpublished health and welfare case files (n 135), [26–37].

155 See also Ruck Keene and others (n 3), [69] where the authors mention that P was only an applicant to court for deprivation of liberty safeguard (DOLS) cases. This is noteworthy as DOLS have a specific mechanism to support people to apply. The authors also reference statistics by Series and others (n 135) which further supports this claim.

156 Following a comparative analysis with case files and one author’s experience as a CoP barrister, Ruck Keene and others also found that their cases were broadly representative (n 3). Case has also argued that there was no reason to believe the BAILII judgments were unrepresentative of the courts’ health and welfare caseload. She acknowledged, however, that CoP cases were grossly underreported in its early years, which hampers a historical understanding of the issues we have raised in this article (n 95).

157 This was noted by Ruck Keene and others (n 3). A key benefit of capacity case reviews is that they make disparate legal developments (including developments around judges handling of influence) accessible to a wider audience.

158 Lindsey has combined capacity case reviews alongside unpublished case files, in-depth interviews of social workers and observations of ongoing CoP proceedings (n 95 and 116). These integrative methods not only reduce selection bias concerns but may also offer insights into earlier, unpublished cases. Craigie’s review (n 18) similarly combined a web-based case search with insights from voluntary sector and public body organizations.

